# Factors Associated with Intended Use of a Web Site Among Family Practice Patients

**DOI:** 10.2196/jmir.3.2.e17

**Published:** 2001-05-17

**Authors:** Peggy A Smith-Barbaro, John C Licciardone, Howard F Clarke, Samuel T Coleridge

**Affiliations:** ^1^Department of Family MedicineTexas College of Osteopathic MedicineUniversity of North Texas Health Science Center at Fort WorthUSA

**Keywords:** Internet/utilization, Questionnaires, Surveys, Patients/statistics, numerical data, Attitude to Computers, Socioeconomic Factors, Age Factors, Health Education/methods, Health Behavior, Family Practice, Logistic Models, Odds Ratio, Digital Divide

## Abstract

**Background:**

The World Wide Web has become a widely utilized source of health information. Although the frequency of health related queries is impressive, the demographics associated with patients making queries has not been clearly delineated.

**Objective:**

This study's objective was to determine health related Internet usage patterns of family medicine patients.

**Methods:**

Internet use among 824 eligible patients 18 years or older attending seven university based family practice clinics during a two week period in November of 1999 was studied. The survey instrument included 10 items and was designed to collect data in less than five minutes using a paper and pencil format. Statistical significance associated with intended Web site use was computed using a multiple logistic regression model.

**Results:**

A response rate of 72.2% was observed with 63.1% being females and 36.9% being males. The mean and median age were 44.0 and 45.7 years, respectively. A steady decline in intended Web site use was observed with advancing age with significant differences observed above 65 years (OR = 0.30; 95% CI = 0.14 - 0.64; p< .002). Other significant findings associated with intended use of a Web site by clinic based patients included having a home computer (OR = 1.99; 95%, CI = 1.05 - 3.76; p<0.03) and having Internet access at home (OR=5.6, 95%, CI = 2.83-11.18; p<.001). A lack of association between intended Web site use and health insurance status was observed.

**Conclusions:**

Results from this study suggest that Web sites are not likely to be alternative sources of health information for the uninsured or elderly in the near future.

## Introduction

Sophisticated consumer health information systems, supported by emerging technologies, are expected to become integral components of future medical practice [1]. Online resources have created not only the potential for health care providers to complement their usual delivery of services, but also to fundamentally alter how they deliver health care [2]. Increasingly, health information and consultations are being sought online, [3,4] with little evidence of international barriers [5]. In fact, it is postulated that online health communications may replace a substantial amount of health care now delivered in person [6].

Nevertheless, questions have been raised regarding access to and the quality of online health information [7]. Among the potential barriers to this emerging resource are cost, location, illiteracy, and disability [8]. Demand for online health information appears to be enormous, with allegedly 25% of search engine queries on the World Wide Web involving health issues [9]. However, it has also been shown that the majority of Medline searches via free Web access are still made by health care professionals and researchers rather than members of the general public or students [10]. In fact, 5% of outpatients at an urban medical center used the Internet to access health information and only 20% reported access to the Internet [11]. Further, a systematic review of computerized educational interventions found that they appear to be a valuable supplement to, but not a substitute for, face-to-face time with physicians [12,13]. To further study the potential of providing online health information in family practice, we conducted a survey of patients attending a network of university clinics.

## Methods

Patients at seven family practice clinics affiliated with the University of North Texas Health Science Center at Fort Worth were surveyed. Six of the seven clinics were located in the Fort Worth/Tarrant County metropolitan area. The seventh clinic was located in a rural, health professional shortage area of an adjacent county. The number of surveys allocated to each clinic was in proportion to its patient population. The survey instrument included 10 items and was designed to collect self-reported data in less than five minutes using a paper and pencil format (Textbox 1). Clinic personnel were trained in survey administration and collection. Eligible patients included those 18 years of age or older who attended one of the participating clinics during a two-week period in November 1999. The survey sought information on patient sociodemographic characteristics, home computer availability, and Internet access. There were no financial incentives for survey participation.

**Survey Instrument**

1. Age ____

2. Sex

female  male

3. Are you:

single  married  other

4. Do you have children

yes  no

5. If you aswered YES please list their age(s)

____ ____ ____ ____ ____ ____

6. Are you

privately insured

a member of an HMO/PPO

uninsured

Medicaid/Medicare

7. Do you have a computer at home?

yes  no

8. Do you have access to the internet?

At home:  yes  no

At work:  yes  no

9. If you aswered YES, how much time do you spend on the internet?

daily __________ weekly__________

10. Would you use a free health information web site provided by the University of North Texas Health Science Center at Fort Worth?

use frequently

use occasionally

Not use

Survey results were summarized using standard descriptive statistics. The main outcome measure was response to the following survey item: " *Would you use a free health information Web site provided by the University of North Texas Health Science Center at Fort Worth*." Responses to this item included " *use frequently*," " *use occasionally*," or " *not use*." A multiple logistic regression model was used to compute odds ratios (ORs) and 95% confidence intervals (CIs) associated with intended use of the clinic-based Web site for each of seven variables while simultaneously adjusting for the other variables. The dependent variable in this model was dichotomized as intended Web site use (either frequent or occasional) vs. no use. Analyses were conducted using the SYSTAT 7.0 for Windows software (SPSS Inc., Chicago, IL). All hypotheses were tested at the .05 level of statistical significance.

## Results

A total of 595 (72.2%) of the 824 eligible patients provided survey information. Patient responses are summarized in Table 1. There was an adequate representation of all age groups among the respondents. A total of 226 (39.0%) respondents had a home computer and 179 (32.1%) also had Internet access at home. There were 242 (48.1%) respondents who stated they would use the clinic-based Web site to acquire health information.

**Table 1:** Sociodemographic characteristics of survey respondents

Variable	No	%
**Age, yr**		
< 35	204	34.7
36-50	153	26.0
51-65	115	19.6
> 65	116	19.7
**Gender**		
Female	370	63.1
Male	216	36.9
**Marital Status**		
Single	251	43.7
Married	307	53.4
Other	17	3.0
**Have Children**		
No	151	26.1
Yes	427	73.9
**Health Insurance**		
None	31	5.4
Private	89	15.6
Health Maintenance Organization /Preferred Provider Organization	355	62.4
Medicare or Medicaid	141	24.8
**Have Home Computer**		
No	354	61.0
Yes	226	39.0
**Have Internet Access at Home**		
No	378	67.9
Yes	179	32.1
**Intended to Use the Clinic-Based Web Site**		
No	261	51.9
Occasionally	166	33.0
Frequently	76	15.1

The multivariate factors associated with intended use of the clinic-based Web site are presented in Table 2. A majority of younger respondents would use the Web site; however, there was a steady decline in intended Web site use with advancing age. Respondents greater than 65 years of age were less likely than young adults to report intended Web site use, even after adjusting for potential confounders such as having a home computer and Internet access (OR=0.30, 95% CI=0.14-0.64; P=.002). Other factors such as gender, marital status, having children, and having health insurance were not significantly associated with intended Web site use. A total of 569 patients responded to the item on health insurance; 47 patients reported multiple insurance types. Almost three-fourths of respondents with a home computer would use the Web site. Having a home computer almost doubled the likelihood of using the Web site (OR=1.99, 95% CI=1.05-3.76; P=.03). Not surprisingly, however, having Internet access at home considerably enhanced the likelihood of using the Web site (OR=5.62, 95% CI=2.83-11.18; P<.001).

**Table 2:** Factors associated with intended use of a Web site by family practice clinic patients

	No. of Users	Total No	% Users	Odds Ratio	95% Confidence Interval	P
**Age, yr**						
< 35*	102	180	56.7	1.00		
36-50	72	132	54.5	0.91	0.51, 1.63	.76
51-65	47	97	48.5	0.69	0.36, 1.33	.27
> 65	19	88	21.6	0.30	0.14, 0.64	.002
**Gender**						
Female*	153	314	48.7	1.00		
Male	86	185	46.5	1.11	0.68, 1.81	.67
**Marital Status**						
Single*	107	220	48.6	1.00		
Married	126	258	48.8	0.88	0.54, 1.45	.62
Other	3	12	25.0	0.76	0.15, 3.85	.74
**Have Children**						
No*	70	128	54.7	1.00		
Yes	167	363	46.0	1.34	0.75, 2.40	.33
**Have Health Insurance**						
No*	11	28	39.3	1.00		
Yes	227	459	49.5	1.19	0.48, 2.96	.71
**Have Home Computer**						
No*	85	282	30.1	1.00		
Yes	155	214	72.4	1.99	1.05, 3.76	.03
**Have Internet Access at Home**						
No*	91	306	29.7	1.00		
Yes	139	171	81.3	5.62	2.83, 11.18	<.001

## Discussion

This study indicates that less than 40% of family practice patients attending a network of university clinics have a home computer and less than one-third have Internet access at home. At most, about one-half of this patient population would use a clinic-based Web site to acquire health information. Slightly higher results were previously reported in a study of primary caregivers of pediatric patients or patients aged 16 years or older. Results from this study showed that 58.9% of study participants reported having a computer or some type of Internet connection [14].

By far, the strongest independent factor associated with intended Web site use was having Internet access at home. (Table 2) We observed that 32.1% of survey respondents have Internet access at home (Table 1). This figure is within the range of previously reported results from a 1999 study of Internet access of genitourinary patients (range 31% - 52%) [15].

Simply having a home computer was not as strongly associated with intended Web site use as having home Internet access. These results suggest that Internet access outside the home, such as at the workplace or at public libraries, may not be conducive to accessing health information, although a variable explicitly representing such access was not actually included in the model. One possible explanation for this phenomenon is that Web site users may prefer the privacy of their own homes and computers in accessing potentially sensitive health information. Almost half of genitourinary patients reported difficulty accessing Internet sites with privacy [15]. This may reflect real or perceived intrusions such as viewing a user's computer display screen or even the possibility of electronic monitoring of a user's trail of Web sites accessed in cyberspace [16,17].

Patients greater than 65 years of age were less likely to report intended Web site use, even after adjusting for such factors as having a home computer and Internet access. (Table 2) This finding indicates that older patients may be more resistant to non-traditional modes of receiving health information and care, or perhaps less educated or interested in computer usage. It is also possible that diseases and functional impairments in the elderly may limit their ability to access and view Web-based health information [18]. This may represent an important barrier in making online health communication an integral part of future health care delivery for chronic, debilitating conditions.

Characteristics such as gender, marital status, and having children were not associated with intended Web site use. These findings, particularly with regard to marital status and children, suggest that intended Web site users may be more interested in acquiring information about personal health matters rather than those of spouses or children. The lack of an association between intended Web site use and health insurance status suggests that Web sites are not currently an important alternative source of health information for those not having health insurance.

There are several limitations of the present study that should be noted. Although the possibility of selection bias among respondents cannot be dismissed, the relatively high response rate achieved in the survey helps minimize its likelihood. However, our results should be extrapolated to other clinic populations with caution because it is unlikely that our survey respondents are representative of the general population, or even of family practice patients in other health care settings. There are also limitations attributable to the survey instrument. For example, data were self-reported and not verified in any manner. Further, there is additional uncertainty because we asked about intended use of a hypothetical Web site rather than current or past use of an existing Web site. Finally, to minimize potential barriers to survey response, we elected not to collect potentially sensitive information such as race/ethnicity, educational level, and income.

It has been noted that the focus of traditional medical informatics is shifting from health professionals to patients, a trend which coincides with the desire of most patients to assume greater responsibility for their health, with the emphasis on public health and prevention [18]. The results of this study indicate that more pervasive Internet access at home is needed to facilitate the public health approach to health information and that barriers to using the Web among older patients must be overcome if they are to become more proactive partners in family practice.
